# Family medicine as a discipline in South Africa: Historical perspectives

**DOI:** 10.4102/jcmsa.v1i1.43

**Published:** 2023-11-30

**Authors:** Indiran Govender, Olufemi Omole

**Affiliations:** 1 Department of Family Medicine and Primary Health Care, School of Medicine, Sefako Makgatho Health Science University, Pretoria, South Africa; 2 Department of Family Medicine, Faculty of Medicine, University of the Witwatersrand, Johannesburg, South Africa

Family medicine is a generalist discipline that provides comprehensive, continuous, evidence-based, first contact and person-centred healthcare, irrespective of the age, gender, diagnosis, special investigation required or personal characteristics of the person, using family-oriented care approach and principles.^1^ This editorial provides a historical perspective of the evolution of family medicine as a discipline in South Africa (SA).

Prior to its recognition as a specialty in 2007, family medicine had undergone many changes, challenges and uncertainties similar to the political turmoil of SA. The two major political events in SA in the 20th century impacted the development of family medicine.^2^ The first was the 1948 takeover by the National Party, which with the support of some members of the medical fraternity put a stop to the promising development of Community-Oriented Primary Care (COPC) project started in Pholela by Sidney and Emily Kark^3^ and of the implementation of community-based primary health care (PHC) proposed by the Gluckman Report. Consequently, nearly a half century passed in which PHC and community-based care were neglected in favour of healthcare services in large hospitals and tertiary centres. During this time, general practitioners (GPs), medical officers and missionary doctors developed PHC care largely without policy support from the government. In many rural areas and socioeconomically deprived areas, GP and missionary doctor-based services predated the emergence of family medicine as a distinct academic discipline.^4^ Doctors in these areas offered basic care at small hospitals and soon extended care to communities through health promotion, health education and PHC clinics. This became a strong rural health movement through the Rural Doctors Association of Southern Africa, the academic rural health units at university medical faculties and the Rural Health Initiative established by the Academy of Family Medicine. In urban areas, there were primary care services such as the day hospitals in the Cape Peninsula and PHC clinics in Soweto, where primary care professionals provided services to large populations. Many of these services became integrated into urban health districts and in recent times form part of the academic training complexes for family medicine. It is important to document that the 1976 Soweto uprising triggered the training of nurse clinicians and made the PHC a shared space for nurse clinicians and GPs, albeit with its own challenges, advantages and implications for PHC services and family medicine training.^3^ Thus, the concepts of COPC and outreach activities were implemented in South Africa much earlier than they became established globally. The second major event, the establishment of democracy in SA in 1994, puts the health policy of the African National Congress in place. This policy is premised on the district health system being the vehicle for the delivery of health care services, prompting family medicine in SA to claim the health district as its domain for clinical practice and decentralised training.^4,5,6^

From the late 1950s, GPs, some with training in the UK, started to organise themselves around academic issues. These were involved in the establishment of the College of General Practice and the Academy of Family Practice and Primary Care. Their main goal was to teach GPs how to reflect on their own practices and to promote learning and research through continuous medical education and vocational training (VT). The South African College of General Practitioners was established on 01 June 1969, and in 1970, it became the Faculty of General Practice of the College of Medicine of South Africa. Following a dispute over the faculty’s autonomy, a new independent body was registered in August 1980 – the South African Academy of Family Practice and Primary Care (SAAFP). General practitioners, however, remained within the College of Medicine (CMSA), which continues as the examining body for the discipline. In 1994, the Faculty of General Practice changed its name to the College of Family Practitioners (CFP). The CFP has the responsibility for the fellowship, diploma and certificate examinations and is governed by a triennially elected council. Recent reforms in composition have resulted in the co-opting of additional members to ensure that all university departments and/or divisions of family medicine are represented, particularly in the examination writing groups.^5^

Dr Basil Jaffe was the first president of the SAAFP, an entity focused on ‘*education, standards of training and the provision of primary care to all the people of South Africa*’. The SAAFP is also governed by an elected council. In 1983, the SAAFP identified a shortage of doctors in rural areas and proposed that doctors be appointed to vacant posts as trainees. This acted as a stimulus to the development of national VT in family medicine and the formation of the Academy Education Committee in 1985 to design, implement, coordinate and monitor the educational programmes. It convened a series of national teacher training workshops between 1985 and 1990, and the first VT programme commenced in Natal in 1985. In 1986, the committee prepared a document entitled ‘Blueprint for vocational training in Family Medicine in South Africa’, which was presented to the South African Medical and Dental Council in 1987.^4,5,6^

With compulsory continuous professional development (CPD) in 2000, the SAAFP became the most active accreditor of CPD in SA. The academy also stimulates interest in research and publication through the *South African Family Practice Journal*, participation in the WONCA and the annual national family practitioners congress.^5,6^

Legislation was enacted in 1993 setting up a register for family physicians. Registration was open to individuals who have completed a recognised family medicine VT programme resulting in a Master of Medicine degree in family medicine or Membership of the College of Family Practitioners (MCFP) or both. In alignment, the public service established rankings of senior, principal and chief family practitioners and physicians as career paths for those who had undertaken the postgraduate VT in the field. Consensus decisions on specific outcomes-based training for general practice and/or family medicine include mandatory postgraduate training for registration for independent practice in family medicine; only one category for generalist doctors named ‘Family Physicians’, which should be a specialty on the specialist register of the HPCSA, and those in training should be named registrars. On 08 May 2003, the senate of the CMSA approved the establishment of a Fellowship degree for the CFP, making family medicine equal on academic footing with other specialist disciplines. From January 2005 on, interns are also required to undergo a 2-year internship that includes a compulsory 4-month rotation in family medicine. In 2007, family medicine was accorded a 4-year registrar training period.^4,5,6^

The Inter-University Centre for Family Medicine Training (ICHO), a partnership between South African and Belgian Units of Academic Family Medicine, through a grant, from the Belgian government, assisted Family Medicine Educational Consortium (FaMEC) with setting up district-based training platforms from August 2003. A national co-ordinator was appointed to conduct inspections of all training sites. The train-the-trainer (TCT) workshops that were implemented are now under the guidance of the SAAFP and in the recent past have obtained funding from the Royal College of General Practitioners (United Kingdom [UK]). Although the statutory bodies and academic family physicians were consulted, there has not been adequate consultation with the government on the direction of family medicine training. Also, generalists in private practice were left out of the discussions until recently. Most of the proponents of having only one title of ‘Family Physician’ for all generalists and compulsory postgraduate training for independent generalist practice have been university-based academics. All these resulted in poor buy-in by stakeholders and stalled some of the developmental aspirations. Unfavourable economic environment, attrition of the generalists, poor working conditions in the public health sector, high doctors’ emigration rates and difficulties in obtaining consensus because of numerous independent practitioner organisations and professional bodies, which all purported to represent the views of generalists.^4,5,6^

Prior to 1958, there were no academic departments of general practice in SA, but doctors in general practice were largely represented in the Medical Association of South Africa. The first academic general practice organisation was formed in 1958, after a visit by Dr. Ian Grant, the distinguished second President of the Royal College of General Practitioners, UK. Similar initiatives also took place in the Orange Free State, Eastern Cape and Natal. The central aim of the South African faculties was to raise and maintain the standard of general practice and/or family medicine through education. Thereafter, the first appointment of a professor of family medicine in 1967 at the University of Pretoria was made, followed by the establishment of the Family Medicine Education Consortium (FaMEC) in 1980. The departments of Family Medicine at the Medical University of South Africa (MEDUNSA) followed suit in 1977 and the University of the Orange Free State in 1978. These departments were key in defining the theory and practice of family medicine in SA and their collaborations led to the establishment of FaMEC, which provided a convergence point for the development of family medicine as a specialist discipline.^5,6^

The history of COPC documents how Sidney and Emily Kark, together with Edward Jali and others, are contributed to the establishment of PHC in SA in the 1940s. These initiatives were explicitly linked with the establishment of academic family medicine in SA. In the early 1950s, as part of the establishment of the Natal Medical School, the then Dean convinced the faculty board to establish Family Practice and Community Medicine as a clinical division. When the Natal Medical School opened in 1956, the name was changed to the ‘Department of Social, Preventative and Family Medicine’ and Sidney Kark was appointed as the first professor and head of department. The clinical training was based on the COPC model akin to what is currently called ‘decentralized training platform’. Sadly, both the Department of Social, Preventative and Family Medicine at the University of KwaZulu-Natal and the Institute of Family and Community Health (IFCH) were dissolved owing to complex national and professional politics prevalent in the 1950s and early 1960s, which were profoundly antagonistic to such innovative approaches.^4,5,6^ However, in post-apartheid South Africa, COPC is now core to Family Medicine training and the continuum of providing individual and population healthcare.

Since inception, generalists in SA have collaborated with external entities. The first training in family medicine in SA was as chapters of the British Royal College of General Practitioners. Links with family medicine in Holland, North America and especially with Ian McWhinney from Canada significantly shaped the early years of the development of the academic family medicine in SA. Later collaboration with Belgian (Flemish) family physicians through the Inter-University Collaboration for Training in Family Medicine (ICHO) helped in the formation of FaMEC. The Belgians funded the ‘Optimisation of Family Medicine Training in SA’ project that ran from 2003 to 2006 and facilitated the standardisation and coordination of family medicine training and development of training complexes, nationally. This project is rolled out to other African countries by SA for the development of family medicine in the sub-continent.^5,6,7^

Several recent initiatives that are creating history include the standardisation of training unit standards and outcomes, restructuring of the unitary exit fellowship examination (Fellowship of the College of Family Physicians of South Africa [FCFP {SA}]), transition to a common national portfolio aligned to entrustable professional activities and workplace-based assessments, development of peer support group for newly qualified family physicians and the creation of a PhD network to promote PhD enrolments. However, much still needs to be done in optimising clinical practice and skills, research engagement and publication outputs and increasing the impact and visibility of family medicine.

In conclusion, the history of family medicine has been stormy but impactful regarding the development of PHC in SA. It has influenced how the theory and practice of family practice are constructed and taught to trainees. As we marvel at how visionary the early generalist healthcare practitioners in SA were, so will future generations do, when they reflect on our own footprints.
